# Smart Contract Vulnerability Detection Based on Deep Learning and Multimodal Decision Fusion

**DOI:** 10.3390/s23167246

**Published:** 2023-08-18

**Authors:** Weichu Deng, Huanchun Wei, Teng Huang, Cong Cao, Yun Peng, Xuan Hu

**Affiliations:** 1 Institute of Artificial Intelligence and Blockchain, Guangzhou University, Guangzhou 510006, China; 2112106114@e.gzhu.edu.cn (W.D.); huangteng1220@gzhu.edu.cn (T.H.); 2112206107@e.gzhu.edu.cn (C.C.);; 2 School of Beidou, Guangxi University of Information Engineering, Nanning 530299, China; 3 Information Security Research Center, CEPREI Laboratory, Guangzhou 510610, China; 4 Key Laboratory of Ministry of Industry and Information Technology, Guangzhou 510610, China

**Keywords:** multimodal fusion, smart contract, vulnerability detection, deep learning

## Abstract

With the rapid development and widespread application of blockchain technology in recent years, smart contracts running on blockchains often face security vulnerability problems, resulting in significant economic losses. Unlike traditional programs, smart contracts cannot be modified once deployed, and vulnerabilities cannot be remedied. Therefore, the vulnerability detection of smart contracts has become a research focus. Most existing vulnerability detection methods are based on rules defined by experts, which are inefficient and have poor scalability. Although there have been studies using machine learning methods to extract contract features for vulnerability detection, the features considered are singular, and it is impossible to fully utilize smart contract information. In order to overcome the limitations of existing methods, this paper proposes a smart contract vulnerability detection method based on deep learning and multimodal decision fusion. This method also considers the code semantics and control structure information of smart contracts. It integrates the source code, operation code, and control-flow modes through the multimodal decision fusion method. The deep learning method extracts five features used to represent contracts and achieves high accuracy and recall rates. The experimental results show that the detection accuracy of our method for arithmetic vulnerability, re-entrant vulnerability, transaction order dependence, and Ethernet locking vulnerability can reach 91.6%, 90.9%, 94.8%, and 89.5%, respectively, and the detected AUC values can reach 0.834, 0.852, 0.886, and 0.825, respectively. This shows that our method has a good vulnerability detection effect. Furthermore, ablation experiments show that the multimodal decision fusion method contributes significantly to the fusion of different modalities.

## 1. Introduction

A smart contract is the technical core of blockchain 2.0 [1], first proposed by computer scientist Nick Szabo in 1995. It is a contract clause established in digital form, with self-verification and automatic execution characteristics. Unlike traditional contracts, smart contracts have high efficiency, low costs, and high security, and no “denial” occurs. Smart contracts are designed to perform contracts securely and efficiently without a trusted third party. Previously limited by technological developments, smart contracts have been widely used with the rapid development of blockchains in recent years. Ethereum [2], as the first blockchain platform to introduce smart contracts, supports the Turing-complete programming language Solidity [3].

In essence, the smart contract is a piece of code that continuously runs on the blockchain [4], which cannot be modified once deployed and is automatically executed when a predetermined condition is triggered. Smart contracts running on the blockchain can achieve reliable information exchange, value transfer, and asset management, so smart contracts are usually bound to assets. Once security problems occur, they can cause substantial economic losses [5]. Smart contract security problems have followed the increase in smart contracts. According to statistics, 89% of smart contracts have security vulnerabilities.

The security of smart contracts [6] has also attracted the attention of hackers, who have been stealing illegal assets by exploiting smart contract vulnerabilities. In 2016, attackers used a re-entrant vulnerability in TheDAO’s crowdfunding contract to attack it, resulting in about USD 60 million in losses [7]. In 2017, a vulnerability in the Parity wallet [8] contract led to USD 31 million in user losses. In 2018, hackers took advantage of the integer overflow vulnerability in the Ethereum ERC-20 smart contract to transfer a large number of BEC tokens issued by the United States Chain company out of thin air, resulting in its market value dropping to almost zero [9]. According to statistics from SlowMist’s website [10], attacks against Ethereum smart contracts have caused cumulative losses of more than USD 3.1 billion as of 2023.

With the frequent occurrence of blockchain security incidents and the increasing number of assets involved in smart contracts, the vulnerability problem of smart contracts has also attracted the attention of more scholars [11]. Due to the characteristics of smart contracts being tamper-evident and automatically executed, stolen assets are difficult to recover once security problems occur in smart contracts. Therefore, there is an urgent need to detect potential security vulnerabilities before deployment to ensure the security of smart contracts and the safety of the asset interests of each participant.

Current smart contract vulnerability detection methods [12] are mainly based on traditional vulnerability detection methods of conventional programs (such as C++ and Java), usually using static analysis [13] and dynamic execution techniques [14] to detect vulnerabilities. This method often relies on expert experience and has problems such as low automation, low efficiency, and insufficient flexibility. In recent years, with the rapid development of deep learning, the use of deep learning technology for vulnerability detection of smart contracts has become a research hotspot [15]. Detection technologies based on deep learning can make up for the problems of low automation, low efficiency, and dependence on expert knowledge in traditional detection methods. However, most of the current methods based on deep learning [16] consider a single data feature and are unable to fully mine the vulnerability information of smart contracts. Additionally, there is poor scalability and a lack of consideration for multimodal characteristics.

### 1.1. Goals and Contributions

In order to address the above challenges, this paper proposes a smart contract vulnerability detection method based on deep learning and multimodal decision fusion, which does not rely on expert knowledge and supports a wide range of vulnerability types. Firstly, multiple features that can be used for vulnerability detection are extracted from smart contracts. In this paper, the source code (SC) [17], operation code (OP) [18], and control-flow graph (CFG) [19] of smart contracts are extracted. Secondly, the extracted features are used to train the neural network classifier, and the decision of each modal data is obtained. Finally, the decisions of these classifiers are integrated with the stacking-decision fusion method as the final decision. This paper experimentally evaluates the proposed approach on a publicly available dataset for four common vulnerability types. The experimental results show that, compared with existing methods, the proposed method can achieve higher accuracy and a higher AUC value, and better affects the vulnerability detection of smart contracts. The approach presented in this paper covers a more comprehensive type of smart contract vulnerabilities. The main contributions of this paper include:

- The proposal of a multimodal decision fusion vulnerability detection method for smart contract vulnerability detection. Experiments show that the proposed method is superior to other methods in detecting smart contract vulnerabilities.
- A comparison of the effectiveness of three modalities—source code, operation code, and control-flow graph—on the vulnerability detection task, revealing the effectiveness of the different modalities.

### 1.2. Layout

This paper is organized as follows. Section 2 reviews the existing research, including traditional vulnerability detection techniques for smart contracts, deep learning-based vulnerability detection techniques, and multimodal fusion techniques. Section 3 describes the flow of the proposed detection method for multimodal decision fusion vulnerability. Section 4 presents the experiments and conclusions. Section 5 discusses the experimental results and the advantages and disadvantages of the proposed method. Section 6 summarizes the work in this paper and presents the conclusion.

## 2. Review

This section compares and analyzes existing smart contract vulnerability detection and multimodal fusion techniques, identifies the advances and shortcomings of the current research, and further elaborates on the advantages of our approach.

### 2.1. Vulnerability Types

Smart contracts, as a piece of program code, are inescapably vulnerable, and these vulnerabilities continue to increase. The SWC vulnerability repository [20] alone has flagged as many as 136 types of vulnerabilities. No single tool can cover all the known and unknown vulnerabilities that may appear. Therefore, to verify the effectiveness of our proposed approach, we experimentally validate it against several common and damaging vulnerabilities. These include arithmetic [21], re-entrant [22], transaction-ordering dependence [23], and locked-ether [24] vulnerabilities. In this paper, these vulnerabilities are, respectively, abbreviated as follows for ease of representation: ARTHM, RENT, TimeO, and LE.

- ARTHM: Smart contracts are usually executed by virtual machines (such as EVMs), where integers have a range of maximum and minimum values that they can represent in the virtual machine. When an integer exceeds this range, an overflow occurs. For example, if a number is stored as a uint8, it means that the number is stored as an 8-bit unsigned number with values ranging from 0 to $2^{8}-1$. Hackers exploit the integer overflow vulnerability and make it easy for ordinary developers to make mistakes when writing smart contracts, resulting in integer overflow in smart contracts.
- RENT: In Ethereum, since smart contracts can call external contracts or send ether, these operations require the contract to submit external calls. Attackers can use these calls outside the contract to cause attack hijacking, allowing the attacked contract can be re-executed at any location. This bypasses the restrictions in the original code, resulting in re-entrant attacks. A re-entrant vulnerability triggered the DAO attack [25]. Ethereum was forced to implement a hard fork to recover the damage, and due to the anonymity of the Ethereum network, the attackers are still at large.
- TimeO: A race condition vulnerability occurs when the outcome of a contract’s operation depends on the order of transactions submitted to it. In Ethereum, transactions are grouped into blocks, and the block confirmation time is approximately 17 s. After receiving the transaction request, the miner selects the transaction to be included in the block based on who has paid a high enough gas price. So, miners can anticipate which transactions will occur before they are finalized. For example, in a smart contract of the information reward type, the contract will reward the person who submits the answer first. In this case, a malicious node running an Ethereum client intercepts the reward by submitting a request at a higher price after receiving a transaction request from another node.
- LE: A locked-ether vulnerability means that ether is at risk of being frozen. The reason for ether locking is that smart contract developers do not consider the transfer function of ether when developing smart contracts. They only consider the receive function of ether. This will result in the smart contract account being able only to receive ether and not being able to transfer it out, resulting in the received ether becoming frozen.

### 2.2. Smart Contract Vulnerability Detection

From the perspective of technology-driven development, smart contract vulnerability detection can be divided into traditional methods and deep learning methods, where traditional methods are driven by expert experience in development. In contrast, deep learning methods are driven by data.

#### 2.2.1. Traditional Methods

Traditional methods for detecting vulnerabilities in smart contracts draw heavily from conventional methods for detecting vulnerabilities in programming languages such as C\C++ and Java, which typically use static analysis and dynamic execution techniques to detect vulnerabilities. For example, Maian [26] uses a symbolic execution approach, which detects vulnerabilities by exploring the invocation paths of the contract; however, the number of paths to be explored grows exponentially with the branching state. Securify [11] and Zeus [27] propose a formal verification approach to vulnerability detection, achieving a low false positive rate of detection; however, these methods rely on expert auditing. ContractFuzzer [28] uses fuzzy testing for vulnerability detection and can successfully detect the RENT vulnerability and Parity wallet vulnerability that led to the DAO contract event.

Although traditional vulnerability detection methods are still widely popular today, they generally suffer from low automation, low efficiency, and long detection times due to their reliance on expert experience, and detecting smart contract vulnerabilities is still time-consuming and labor-intensive.

#### 2.2.2. Deep Learning Methods

In recent years, with the rapid development of deep learning, vulnerability detection of smart contracts using deep learning techniques has become a hot research topic. Based on the data-driven deep learning approach, the key to the effectiveness of vulnerability detection for smart contracts lies in the extraction of features and the amount of data used for training. For example, Gao et al. [29] proposed an automated method based on word embedding to learn the features of smart contracts in Solidity. Zhang et al. [30] proposed a vulnerability detection method that combines information graphs and integrated learning using information graph embedding to extract features from smart contracts. Sendner et al. [31] proposed the first migration learning-based vulnerability detection method for smart contracts, which uses a generic feature extractor to learn the bytecode semantics of smart contracts and uses independent branches to learn the features of each vulnerability type. Zhuang et al. [32] first proposed a contract graph to represent the syntactic and semantic structures of smart contracts and then used graph convolutional neural networks to analyze vulnerabilities built on top of the contract graph.

Vulnerability detection technologies based on deep learning can address the problems of low automation, low efficiency, and dependence on expert knowledge of traditional detection methods. However, most of the current methods based on deep learning consider limited data characteristics, are unable to fully mine the vulnerability information of smart contracts, have poor scalability, and lack consideration for multimodal characteristics.

### 2.3. Multimodal Fusion

A modality is a description of something from a particular perspective. Multimodality usually contains two or more modalities and refers to a description of things from multiple perspectives. Usually, to utilize data from multiple modalities, it is necessary to fuse these modal data. According to the different fusion stages, multimodal fusion techniques can be divided into feature-based, decision-based, and hybrid fusion approaches. Currently, multimodal data fusion is one of the most effective ways to efficiently utilize large amounts of multisource data.

#### 2.3.1. Feature-Based Fusion

**Figure 1:**
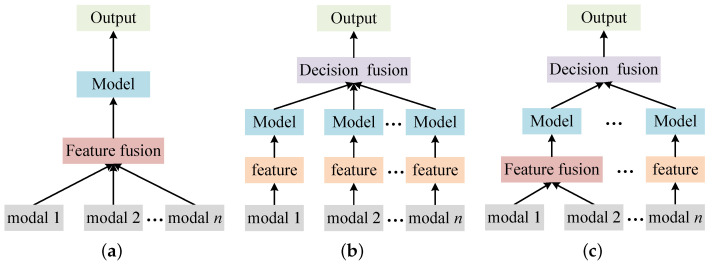
Comparison of the three fusion methods. (**a**) Feature-based fusion. (**b**) Decision-based fusion. (**c**) Hybrid Fusion.

The process of feature-based fusion is shown in Figure 1a. Feature-based fusion usually involves extracting features from different modalities and then fusing them directly, which is suitable for cases where there is a high correlation between modalities. Yang et al. [33] used feature-level fusion for audio and video features for speech recognition, but it suffers from the deficiency of challenging feature extraction. Besides deep learning-based feature extraction, the fuzzy inference method and generative rule method are standard methods for feature-level fusion. The generated high-dimensional feature vector limits feature-based fusion, and the method cannot model complex relationships because of the difficulty in obtaining the cross-correlation between different features by directly fusing multiple modal features.

#### 2.3.2. Decision-Based Fusion

The process of decision-based fusion is shown in Figure 1b. Decision-based fusion [34] considers the variability of different modalities by using the features extracted from different modalities to train sub-models individually After obtaining decisions based on each modality, a combination of these decisions (e.g., vote, sum, average, etc.) is used as the final result. Decision-based fusion is a way to fuse the local decision information obtained from the data after further evaluation or inference [35], and each fusion modality is assumed to be independent of the others. Decision-based fusion is commonly used in human–computer interaction scenarios [36] such as emotion recognition [37]. Makiuchi et al. [38] proposed a new cross-representational speech model for emotion recognition. The model is based on a Transformer model to extract features and uses a CNN model to make decisions. Finally, based on the results from speech and text, the two modalities are fused using a fractional fusion approach, resulting in good outcomes. Xie et al. [39] fully exploited the similarity information between graphical texts and used a multilayer semantic decision-level fusion model to classify the sentiment of graphical texts in social media. Decision-based fusion can fuse heterogeneous data but suffers from the drawback of easy information loss.

#### 2.3.3. Hybrid Fusion

The process of hybrid fusion is shown in Figure 1c. The hybrid fusion approach combines feature- and decision-based fusion. As a compromise between the two fusion methods, the limitations of feature- and decision-level fusion are improved by considering the modality’s characteristics and the learning task’s impact. Lin et al. [40] used feature- and decision-level fusion for audio and video, respectively, applied to a sentiment analysis study, and demonstrated that multimodal fusion outperformed unimodal fusion. Additionally, the decision-level fusion results were slightly better compared to the feature-level fusion results. Although hybrid fusion combines the advantages of feature- and decision-level fusion, it also makes the model more complex and makes learning more difficult.

#### 2.3.4. Discussion

Figure 1 shows the flows of the three fusion methods. Each of the three fusion methods has advantages and disadvantages. Feature-based fusion captures the relationship between features better but is prone to overfitting. Decision-based fusion is less prone to overfitting, and different modalities have less influence on each other, but it does not allow the classifier to train all the data simultaneously. The hybrid fusion method is flexible to use, and while it synthesizes the advantages of both, it also increases the model’s structural complexity and training difficulty.

In real-world smart contract vulnerability detection, the data distribution of vulnerability samples is often unbalanced [40]. On unbalanced datasets, the method based on feature fusion is more likely to overfit. In addition, when it is unclear whether a modality is suitable for smart contract vulnerability detection, the feature-based fusion method needs to consider the impact that adding features of a modality may have on the overall detection performance. In contrast, the decision-based fusion method can use all possible experiment modalities. The correlation between the three modalities of smart contracts used in this paper is small, and all modalities can be independently applied to the vulnerability detection of smart contracts. Therefore, the feature-based fusion method is not considered to avoid interference between different modalities. Last but not least, decision-based fusion methods are more interpretable.

We compared the scope and examples of applications of these three modal fusion approaches. The results of this comparison are shown in Table 1.

## 3. Our Method

This section describes the proposed approach, including the general structure and specific process.

### 3.1. Overview

#### 3.1.1. Structure

**Figure 2:**
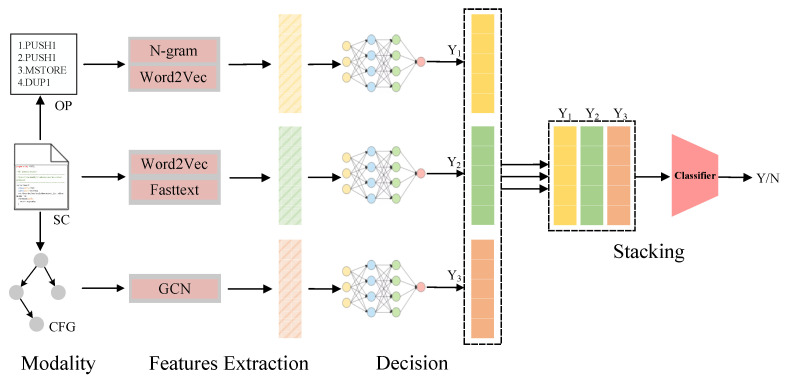
Multimodal fusion vulnerability detection scheme graph. Source code is the initial form in which a smart contract is written out and stored as a sequence of text. The operation code is the product of the compiler’s compilation and records each EVM operation step when executing the contract. The control-flow graph shows how each code block is invoked when the contract is running.

The overall structure of the proposed approach is shown in Figure 2, and the whole process is divided into subclassifier learning and multimodal decision fusion. The subclassifiers make decisions based on the characteristics of the different modalities of the smart contract and determine whether there is a vulnerability. On the other hand, multimodal decision fusion takes each subclassifier’s decision output as input and learns decision fusion strategies based on the decision output and the true labels.

#### 3.1.2. Procedure

**Figure 3:**
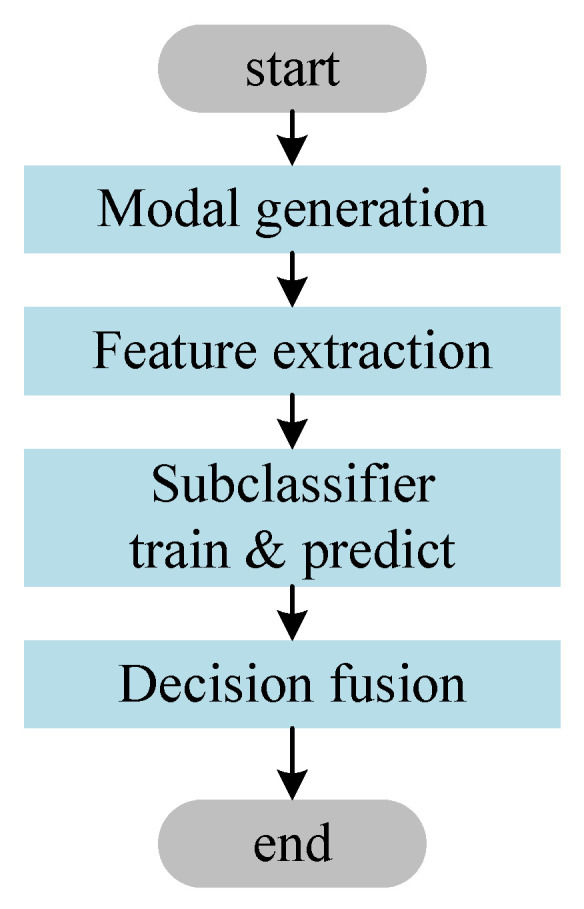
The procedure of our method. Our method is divided into four main stages: modal generation, feature extraction, subclassifier training and prediction, and decision fusion.

As shown in Figure 3, the procedure of our method is divided into four stages according to the order of execution.

1. Modal generation. This stage generates different modal representations of the smart contract data based on the source code. This stage inputs the source code and outputs both the OP and CFG modalities.
2. Feature extraction. This stage extracts different features from the modalities for deep learning model training. This stage inputs the modal data and outputs multiple features.
3. Subclassifier training and prediction. The features extracted from each modality are used to train a deep neural network classifier to make a decision for a particular modality. This stage inputs the features of the modality and outputs the predicted values.
4. Decision fusion. This stage uses the stacking method to fuse the outputs of the individual subclassifiers. A final classifier is trained using the outputs of the subclassifiers as features. This classifier outputs the final prediction.

#### 3.1.3. Discussion

This subsection discusses the feasibility of our proposed methodology. The source code contains the most primitive information about the smart contract [49] and is the primary basis for identifying vulnerabilities [50]. Compared with the source code, the operation code records more detailed operations of the EVM [51] when executing smart contracts, and contract violations can be detected through the operation code. The operation code records semantic information about the contract, whereas the control-flow graph records structural information about the contract. Some vulnerabilities tend to have anomalous structures. Table 2 shows the application of the three modalities for smart contract vulnerability detection.

In summary, the three modalities are feasible for smart contract vulnerability detection.

### 3.2. Modality Generation

The complete life cycle of a smart contract, from writing to execution, can be divided into three layers: the source code layer, build layer, and execution layer. This paper corresponds to these three layers using three modalities for vulnerability detection: source code (SC), operation code (OP), and control-flow graph (CFG). The features of the different modalities of smart contracts are first extracted. Then, different classifiers are trained using these features to make good use of the attributes of a single modality for vulnerability detection. Since the modal data of the smart contract’s operation code and control-flow graph are usually not directly available, the operation code and control-flow graph need to be generated based on the source code.

#### 3.2.1. Operation Code Generation

The operation code records the specific operation of each step of the virtual machine when the smart contract is executed, which is equivalent to a system instruction. Common operation codes and their corresponding meanings are shown in Table 3.

**Figure 4:**
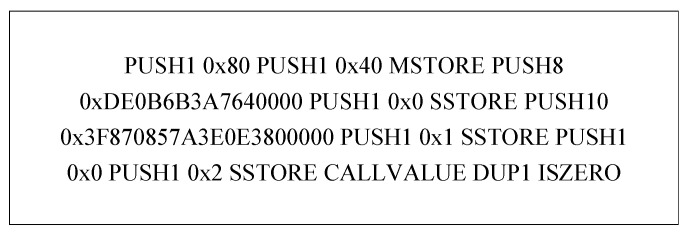
An operation code fragment.

The solc compiler usually generates the operation code for smart contracts. A fragment of operation code generated using the example contract 0x0ce6d5a093d4166237c7a9-ff8e0553b0293214a1.sol is shown in Figure 4.

Given that the meaning of the operation code is independent of the address, the operation code needs to be normalized to prevent the extracted features from having too large a dimension. The processing method used in this paper is as follows:

- The instructions PUSH1-PUSHx, POP1-POPx, etc., are unified as PUSH and POP;
- Remove the operands 0x80, 0x40, 0x0, etc.;
- Remove the operation addresses, such as 0xDE0B6B3A7640000.

**Figure 5:**
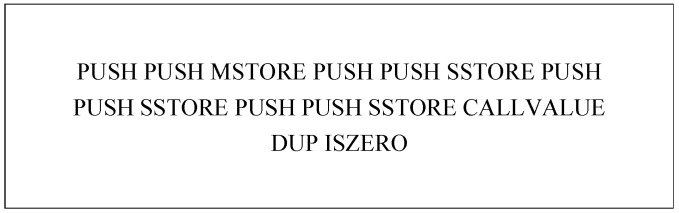
An operation code fragment specification.

The processed operation codes are shown in Figure 5, and the normalized operation codes proceed to the next stage of feature extraction.

#### 3.2.2. Control-Flow Graph Generation

**Figure 6:**
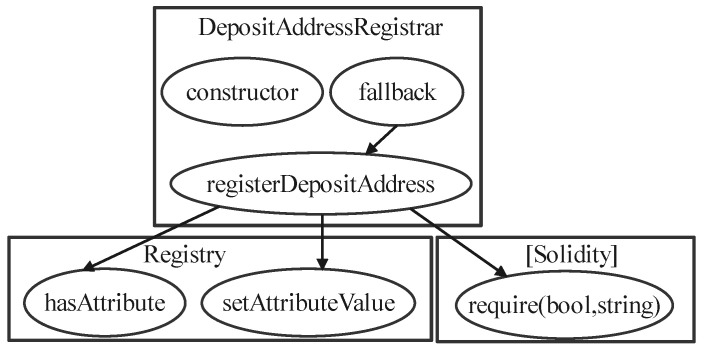
Example of a control-flow graph. The source code for this contract can be found in Appendix A. Combining the control-flow graph with the source code clearly shows the relationship between the code calls.

The control-flow graphs record the variables and functions that each part of the system calls when the EVM executes a smart contract. This paper uses the Slither [60] tool to generate the control-flow graphs. Control-flow graphs generated using the sample contract 0x00000000000da14c27c155bb7c1ac9bd7519eb3b.sol are shown in Figure 6.

As can be seen in Figure 6, the control-flow graph of a smart contract is a kind of directed acyclic graph, where the nodes of the graph are the various functions/variables in the contract, and the edges of the graph represent the invocation relationships between the functions or variables. The control-flow graphs show the structural information of the smart contract.

### 3.3. Modal Features Extraction

In Section 3.2, we obtained three different modal representations of the same smart contract. In this subsection, we extract features from these modalities to obtain a more computationally friendly form and to remove redundant information.

#### 3.3.1. Source Code Embedding

The source code of a smart contract consists of lines of characters that cannot be used directly in a machine learning model and must be transformed into a numerical vector, i.e., text embedding. Keywords and variables with linguistic information from the smart contract in the source code are embedded into the high-dimensional space by performing text embedding on the source code. This paper embeds the source code using the Word2Vec model [61] and the Fasttext model [62].

Word2Vec is the process of mapping a sparse word vector of one-hot form into an n-dimensional dense vector using a single implicit layer, where words with similar meanings are mapped to similar positions in the vector space. Using neural networks allows flexible modeling of context, and it is one of the most popular approaches to text embedding. Word2Vec is based on the idea that the context of a word determines the semantics of that word. According to the semantic decision, Word2Vec is divided into the CBOW and Skip-gram models. Among them, the CBOW model predicts the central word based on the words around it, whereas the Skip-gram model predicts the surrounding words based on the central word.

Fasttext is a Facebook open source word vector and text classification tool that provides a simple and efficient method for learning text classification and representation with high performance and speed. The Fasttext model is a word vector-based text classification model. It uses character-level n-gram-based features to represent words in the text, thus avoiding the traditional bag-of-words model, which must consider all possible word sequences. In Fasttext’s training process, each word is represented as a fixed-length vector. These vectors are combined into a vector representation of the text, and finally, a softmax activation function is used for classification.

The process of embedding source code using the Word2Vec and Fasttext models is as follows. The first step is to clean the text. It may contain various characters unrelated to the code’s semantics (e.g., comments), so the source code text needs to be cleaned before training with the model, including removing deactivated words, comments, and useless punctuation. The next step is model training. After obtaining the text of the source code after text cleaning, the text is processed into a suitable form for the input of the Word2Vec and Fasttext models, and then the model is trained using the text. The final step is text-vector generation. The trained model performs text embedding for all contracts to obtain text vectors containing semantic information and supporting mathematical operations.

#### 3.3.2. Operation Code Vectorization

Before the smart contract runs, the source code must be compiled into the form of operation code, which contains the basic operations of the program’s execution (such as pop stack, push stack, take operand, etc.). Compared to the source code, the operation information contained in the operation code is more detailed and accurate. Similar to the source code, operation code sequences cannot be directly applied to machine learning models. This paper uses simultaneous N-gram and Word2Vec models to vectorize the operation code.

N-gram is an algorithm based on a statistical language model [63]. The basic idea is to process the content within the text using bytes within a sliding window of size N, forming a sequence of byte fragments of length N. Each byte fragment is called a gram, and the frequency of all grams is counted and filtered according to a pre-set threshold to form a list of crucial grams. This list constitutes the vector feature space of this text, with each kind of gram in this list representing a feature vector dimension. In order to prevent the generated vector dimensions from becoming too large, this paper uses a sliding window of N = 2 to extract features from the normalized operand sequence, ultimately generating 5183-dimensional features through experimentation.

The process of vectorizing operand sequences using the Word2Vec model is similar to the embedding process of the source code. First, the text is pre-processed to obtain the training corpus for the Word2Vec model. Then, the Word2Vec model is trained using the corpus, and the text vectors of individual characters are computed sequentially after the training is completed. Finally, the text vectors in all operands are summed to obtain the operand vectors of the whole contract.

#### 3.3.3. Graph Convolution of Control-Flow Graphs

The graph neural network, a deep learning-based graph data processing method with good performance and interpretation, is widely used in various fields. The graph convolution network (GCN) [64] is a feature extractor similar to convolutional neural networks, except that its object is graph data. The graph convolution generally consists of three processes:

1. Send: Each node sends its feature information to its neighbor nodes after transformation. This step involves extracting and transforming the feature information of the nodes.
2. Receive: Each node aggregates the feature information of neighboring nodes. This step involves fusing the local structure information of the nodes.
3. Transform: Perform nonlinear transformation after aggregating the previous information to increase the expressiveness of the model.

In this paper, the features of the control-flow graph are extracted using a graph convolutional neural network, and the final experiment generates 64-dimensional features that are used in later classifier decisions.

### 3.4. Multimodal Decision Fusion

After obtaining the features from the different modalities, a neural network classifier is trained for each modality, and this classifier is used to apply the features of the different modalities. After obtaining the decisions from the neural network for each modality, stacking is applied to fuse the decisions and obtain an optimal decision that considers all the modalities.

The stacking decision fusion uses the output of each subclassifier as input. Suppose the classifiers obtained from each modality training are $\left\{L_{1}\left(x\right),L_{2}\left(x\right),\ldots ,L_{n}\left(x\right)\right\}$, and the corresponding decision outputs are $\left\{y_{1},y_{2},\ldots ,y_{n}\right\}$. Then, the single input of the fusion model is $\left\{y_{1},y_{2},\ldots ,y_{n},Y\right\}$, where *Y* is the true label. Various machine learning models can be used as the decision-fusion model, but this paper uses logistic regression as the fusion model. The reasons are as follows:

- There are not many kinds of modalities, and the dimensionality of the input of the fusion model is not large, so it is not easy to overfit by using simple models.
- The logistic regression model is highly interpretable, and the importance of each modality on the final decision can be obtained to judge the effectiveness of each modality on contract vulnerability detection.

In this paper, we use the binomial logistic regression model. As a classification model, the binomial logistic regression model is represented by a conditional probability distribution P(Y=1∣x). The random variable *X* takes the value of a real number, and the random variable *Y* takes the value of {0,1}. The logistic regression model is defined in Equation (1). In the logistic regression model, the log odds of the output Y=1 form a linear function of the input *x*. In model learning, for a given dataset *T*, the parameters of the model can be estimated using the maximum likelihood method.

(1) $$\log\frac{P(Y=1|x)}{1-P(Y=1|x)}=w\cdot x$$

      In Equation (1), $x\in R^{n+1}$ is the input, Y∈{0,1} is the output, and $w\in R^{n+1}$ is the parameter known as the weight.

The basic idea of using stacking decision fusion in this thesis is to combine the decisions from each modality to form the optimal decision. Assuming that the effect of a particular modality is insignificant and the decision holds no value, it is also possible to set voting weights, etc., so that the final decision of that modality has the lowest impact on the final decision. The worst effect of decision-level fusion will not be lower than the effect of the best single modality.

## 4. Experiments

In this section, we empirically evaluate our proposed approach using a publicly available dataset. In order to evaluate the performance of the proposed method, the following research questions were designed:

- RQ1: Can the proposed method effectively detect common vulnerabilities in smart contracts, and is its vulnerability detection better than that of existing methods?
- RQ2: Does adding new features within the same modality help improve model performance?
- RQ3: Do different strategies of cross-modal fusion affect model performance?

The following experiments address each of these research questions.

### 4.1. Experiment Settings

#### 4.1.1. Dataset

In this section, we conduct tests on a publicly available dataset and analyze the effectiveness of the proposed approach from multiple perspectives. We used the ScrawID dataset [65], a real Ethereum smart contract dataset with vulnerability tags, to test against four common contract vulnerability types: ARTHM, TimeO, LE, and RENT. We employed a crawler to obtain the source code of the corresponding contracts from Ethscan. Considering the unbalanced nature of the data, we resampled a few classes of categories using the ADASYN algorithm [66]. A total of 9252 smart contract data were used in the experiments.

#### 4.1.2. Experiment Environment

The vulnerability detection model proposed in this paper mainly consists of four parts: modal generation, which compiles source code into an operation code and generates a control-flow graph; feature extraction, which is used to extract the features of each mode; a neural network classifier, which is used to classify samples according to each modal feature; and decision fusion, which is used to fuse the output of each modal classifier. The modal generation tools were mainly implemented using Python and SIF tools [67]. Feature extraction was implemented through text embedding and graph convolution models. The neural network classifiers and decision fusion were implemented using Pytorch and SkLearn. The system environment versions included Ubuntu 18.04, Python 3.10, Scikit-learn 1.2.2, and Pytorch 1.13.1.

All experiments were conducted on a computer equipped with an Intel Xeon Gold 6240R CPU @ 2.6 GHz, GPU Tesla V100S-32 GB, and 64 GB RAM.

#### 4.1.3. Parameter Setting

The input type of the graph convolutional neural network contained the following three parts: the dimensionality of each node in the graph (uniformly coded as 128 dimensions in this paper), the representation of the adjacency matrix corresponding to the nodes in the graph, and the label corresponding to the graph. The output was the number of graph categories. The hidden layer was 128×128×128×128×128×64, and the batch size was set to 256. The neural network classifier was a fully connected feedforward network with 200×100×100×50 hidden layers. There is no straightforward method for determining the number of layers of the neural network, and we determined a better layer configuration based on experience and experiments.

The activation function of both networks was Relu, a binary cross-entropy loss function. The Relu activation function is nonlinear and less prone to gradient vanishing. The network parameters were adjusted using the Adam optimizer, which is commonly used in deep learning. The learning rate for the graph convolutional neural network was set to 0.005, and the learning rate of the fully connected feedforward network was set to 0.001. A lower learning rate prevents neural networks from converging to local optimal points.

Decision fusion was implemented using a logistic regression model, and to prevent overfitting, $L_{2}$ regularization was applied to constrain the model parameters. A grid search method was applied to explore the optimal regularization parameters within the range of [0.001, 20], with 500 equidistant points. $L_{2}$ regularization was chosen because there were fewer features, and $L_{2}$ regularization prevents overfitting.

We randomly selected 70% of the dataset as the training set and the remaining 30% as the test set. and report the average results from multiple training runs.

#### 4.1.4. Evaluation Metrics

We applied the following four widely used evaluation metrics to measure the effectiveness of our method against other methods. Accuracy (ACC) is the percentage of all samples correctly detected. Precision (P) is the ratio of correctly detected vulnerable samples to all detected vulnerable samples. Recall (R) is the ratio of correctly detected vulnerable samples to all vulnerable samples. The F1 score (F1) is the summed average of precision and recall, used as a score to measure the overall effectiveness. The AUC value (i.e., the area under the ROC curve) can be used to compare the performance of different classifiers. The exact formulas for the evaluation metrics are as follows:

(2) $$\mathrm{ACC}=\frac{\mathrm{TP}\;+\;\mathrm{TN}}{\mathrm{TP}\;+\;\mathrm{FP}\;+\;\mathrm{TN}\;+\;\mathrm{FN}}$$

(3) $$R=\frac{\mathrm{TP}}{\mathrm{TP}\;+\;\mathrm{FN}}$$

(4) $$P=\frac{\mathrm{TP}}{\mathrm{TP}\;+\;\mathrm{FP}}$$

(5) $$F1=2\times \frac{P\times R}{P\;+\;R}$$

### 4.2. Comparison Experiment (Addressing RQ1)

In order to verify the effectiveness of the proposed method, a comparative test was conducted using existing smart contract vulnerability detection tools on the same dataset. These tools included Mythril [68], Smartcheck [69], and Slither [60]. In addition, we selected TMP [32] as a similar method for comparison. Table 4 shows the comparison of these methods and our proposed method, these methods are described as follows:

- Mythril: A free security analysis tool provided by the Ethereum open source community. Mythril, one of the most well-known smart contract security tools, can detect security vulnerabilities in Solidity smart contracts and perform in-depth analysis. Mythril is a static vulnerability analysis tool that relies simply on symbolic execution [70] and the SMT method to perform vulnerability detection. It currently supports the detection of vulnerabilities including integer-overflow, timestamp-dependency, and re-entry attacks.
- Smartcheck: A scalable static analysis tool for detecting vulnerabilities or code issues in Solidity smart contracts. Smartcheck converts smart contracts into a structured XML form and then identifies smart contract vulnerabilities by matching Xpath patterns. Although Smartcheck is a simple and efficient tool for detecting smart contract vulnerabilities, the types of vulnerabilities supported for detection are limited.
- Slither: The first open source static analysis framework for the Solidity language. Slither can find vulnerabilities within seconds as a tool dedicated to the security analysis of Solidity smart contracts. Slither is user-friendly and provides numerous APIs for developers to use. However, the tool suffers from underreporting of severe issues, such as not being very sensitive to detecting integer-overflow vulnerabilities and requiring manual code auditing.
- TMP: A novel temporal information propagation network that learns vulnerability features in a normalized contract graph using graph convolution. A contract graph is a graph that represents data and control dependencies between program statements.

The comparison experiments were performed on the same dataset using tools including Mythril, Smartcheck, Slither, TMP, and the method proposed in this paper. The experimental results are shown in Table 5. As can be seen from the experimental results, the method proposed in this paper can support more types of vulnerabilities because it does not rely on expert knowledge. Compared to existing mature, smart contract vulnerability detection tools, the method proposed in this paper achieved higher detection accuracy and AUC values on various types of vulnerabilities, as well as better overall detection performance.

The traditional approach relies on expert experience and requires constant updating of the characteristics of smart contract vulnerabilities. Often, even experienced experts make mistakes and omit some vital information that can be used for vulnerability detection, which is the root cause of the poor effectiveness of this type of smart contract vulnerability detection method. TMP is a vulnerability detection method based on the concept of contract graphs that was proposed in [32]. The graph-based detection method is often suitable for security vulnerabilities caused by contract inter-call. In Table 5, it can be seen that TMP achieved the best detection accuracy for the RENT vulnerability. At the same time, vulnerabilities such as ARTHM may appear in a line of code and cannot be represented graphically. Traditional and deep learning-based approaches use limited information about the characteristics of contracts.

Our method uses as much contractual characterization information as possible and combines this information organically. Therefore, our method’s vulnerability detection is better than existing methods.

### 4.3. Ablation Experiments

In this subsection, we verify the effectiveness of each part of the proposed method. Firstly, an experimental comparison between features within a single modality and feature fusion is performed. Secondly, the effectiveness of cross-modal fusion is verified.

#### 4.3.1. Comparison between Features within a Single Modality and Feature Fusion (Addressing RQ2)

After extracting the features from the modalities using the feature extractor, the neural network classifier was trained directly, and the effects were compared. The ARTHM vulnerability was used as an example to compare the effectiveness of different types of features within the different modalities for vulnerability detection, and the experimental results are shown in Table 6.

In Table 6, it can be seen that the addition of new features of a modality can effectively improve the performance of the training model in cases where the detection performance of the original single intra-modal feature is poor.

#### 4.3.2. Comparison of Inter-Modal Features and Multimodal Decision Fusion (Addressing RQ3)

Based on the information in Section 4.3.1, the neural network classifier was trained using the features obtained through intra-modal feature fusion, and the experimental results of the different modalities were compared. Table 7 shows the experimental results of the different modalities for four vulnerability types, where Concat indicates that the features of these modalities were stitched together to form a new feature and used as the input of the neural network classifier. In Table 7, it can be seen that the features of the OP outperformed those of the CFG and SC on the vulnerability detection task. The direct fusion of cross-modal features resulted in a slight performance improvement compared to the optimal modality.

We obtained the decision outputs from each modality, and the results of decision fusion using logistic regression are shown in Table 8. The modalities corresponding to the weights are OP, SC, CFG, and Concat. The magnitude of the weights of logistic regression can be regarded as the importance of the influence of the different modalities on the results, and the positive and negative signs can be interpreted as the decision propensities of the modalities.

**Figure 7:**
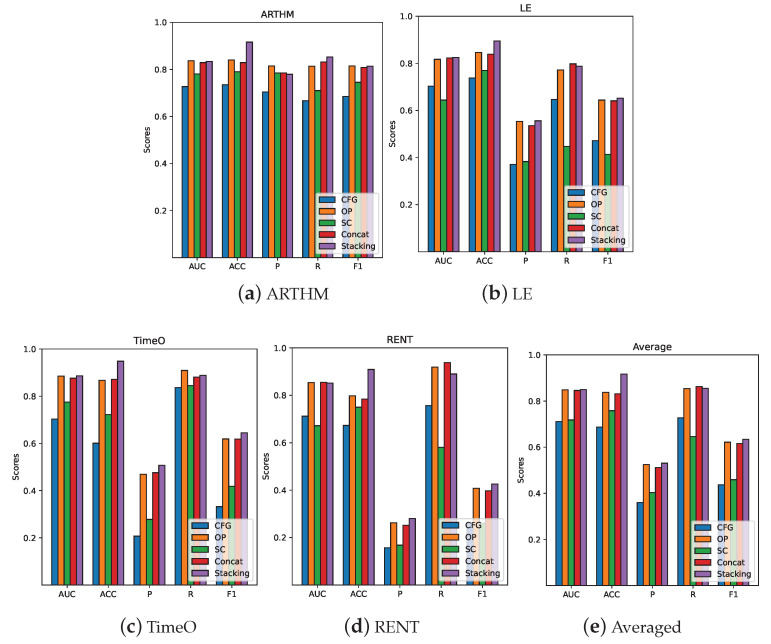
Performance evaluation of different modalities for different types of vulnerability detection. In the figure, the OX-axis represents the different evaluation indicators; the OY-axis represents the score of a particular evaluation indicator, which takes a range of 0∼1. In addition to the AUC, the other indicators can also be interpreted as percentages. (**a**–**d**) Experimental results for different vulnerabilities. (**e**) Averaged results.

As shown in Table 8, the performance improvement of sampling for unbalanced samples was significant, especially for the ACC and AUC values. However, the improvement in the accuracy rate was not significant and can make the model more inclined to classify the samples into a few categories. Figure 7 further visualizes the results shown in Table 7 and Table 8.

The detection method using logistic regression models for multimodal models performed better for the detection task of four common smart contract vulnerabilities. The ACC and AUC values of the decision fusion model improved significantly compared to the method that directly fused the features of each modality.

## 5. Results

The experimental results show that the smart contract vulnerability detection method proposed in this paper outperforms existing methods, achieving high accuracy and a high recall of vulnerability detection. Ablation experiments show that adding new features within the same modality can improve the performance of the vulnerability detection model. In the smart contract vulnerability detection task, the decision fusion of multiple modalities contributes more to improving detection accuracy. In addition, using the resampling method to address the dataset’s unbalanced vulnerability types can somewhat mitigate the imbalanced category problem. However, the detection accuracy rate cannot be improved. In the vulnerability detection task, losses from missed detections are often more significant than those from false positives, so achieving a high recall rate is more important than a high accuracy rate.

## 6. Conclusions

In this paper, from a multimodal perspective, three modalities—source code, operation code, and control-flow graph—with five features were extracted for vulnerability detection from the combined smart contract life cycle. The multimodal decision fusion approach can fully utilize the semantic and structural features of smart contracts. The results of the comparison experiments also verify the proposed approach’s effectiveness and superiority.

Compared with existing smart contract vulnerability detection tools, the proposed approach has many advantages. Firstly, the method does not rely on expert experience and is completely data-driven. Secondly, the method is highly flexible. Finally, the method covers more types of vulnerabilities and achieves a higher inspection accuracy rate. The method proposed in this paper can be used for the initial screening of smart contract vulnerabilities to increase the efficiency of smart contract developers and auditors in identifying smart contract vulnerabilities. Based on this paper, adding new modalities can further improve the performance of vulnerability detection.

Some data imbalances in the experiment led to low detection accuracies. Future work can consider using small samples and unsupervised learning to address this problem. Furthermore, the lack of a standard unified smart contract vulnerability dataset remains an urgent problem that needs to be addressed.

## Figures and Tables

**Table 1:** Comparison of the three fusion methods.

Method	Stages	Scope of Applicability	Examples
Feature-based	Early	Emotion recognition	[41,42,43]
Decision-based	Terminal	Emotion recognition	[44,45]
Hybrid Fusion	Early and Terminal	Event detection	[46,47,48]

**Table 2:** Examples of the three modalities used for vulnerability detection.

Modality	Author	Year	Outcome
	Jiang et al. [52]	2022	High detection performance
Source code	Gao et al. [29]	2020	Semantic clone detection
	Jeon et al. [53]	2021	High detection performance
	Albert et al. [54]	2022	Smart contract optimization
Operation code	Zhang et al. [55]	2023	Short detection time
	Ashizawa et al. [56]	2021	Detects both code clones and vulnerabilities
	Liu et al. [57]	2023	High detection performance
Control-flow graph	Agarwal et al. [58]	2022	High detection accuracy rates
	Liu et al. [59]	2023	Enables fine-grained vulnerability detection

**Table 3:** Operation codes and their meanings.

Type	Name	Meaning
	PUSHx	Push x-byte values on the stack
Stack operations	POPx	Pop x-byte values on the stack
	SWAPx	Swap the top of the stack with the value of the xth position
	AND	Logical operations: AND
Logic commands	OR	Logical operations: OR
	NOT	Logical operations: NOT
	ADD	Arithmetic operations: ADD
Arithmetic commands	MUL	Arithmetic operations: MUL
	SUB	Arithmetic operations: SUB
	ADDRESS	Obtains the address
Address and wallet commands	BALANCE	Obtains the balance
	ORIGIN	Obtains the execution start address of the current contract
	TIMESTAMP	Obtains the timestamp
Predictable variable commands	NUMBER	Obtains the block number
	DIFFICULTY	Obtains the mining difficulty

**Table 4:** Comparison of existing methods and our proposed method.

Method	Year	Technical Category	Vulnerability Types
Mythril	2017	symbolic execution	medium
Smartcheck	2018	static analysis	few
Slither	2019	static analysis	few
TMP	2020	deep learning	many
Our method	-	deep learning	many

**Table 5:** Experimental comparison with other methods.

Method	ARTHM		RENT		LE		TimeO	
	ACC	AUC	ACC	AUC	ACC	AUC	ACC	AUC
Mythril	0.681	0.689	0.716	0.843	-	-	0.879	0.5
Smartcheck	0.564	0.5	-	-	0.925	0.798	-	-
Slither	-	-	0.729	0.851	0.927	0.804	-	-
TMP	0.628	0.561	0.745	0.669	0.841	0.507	0.772	0.684
Our method	0.916	0.834	0.909	0.852	0.895	0.825	0.948	0.886

The “-” symbols in the table mean that the vulnerability detection tool does not support this type of contract.

**Table 6:** Intra-modal compar. Using ARTHM as an example.

Modal	Feature	ACC	AUC	R	P	F1
	FastText	0.741	0.739	0.724	0.674	0.699
SC	Word2Vec	0.839	0.824	0.728	0.871	0.793
	Merge	0.851	0.844	0.787	0.861	0.823
	N-gram	0.858	0.846	0.758	0.898	0.822
OP	Word2Vec	0.847	0.835	0.741	0.887	0.807
	Merge	0.856	0.843	0.74	0.914	0.818
CFG	GCN	0.745	0.733	0.648	0.730	0.687

**Table 7:** Inter-modal comparison.

Type	Use_Data	AUC	ACC	P	R	F1
ARTHM	CFG	0.727	0.735	0.704	0.667	0.685
	OP	0.837	0.840	0.815	0.814	0.815
	SC	0.781	0.790	0.785	0.710	0.745
	Concat	0.829	0.829	0.785	0.832	0.808
LE	CFG	0.703	0.738	0.371	0.647	0.472
	OP	0.817	0.846	0.553	0.772	0.644
	SC	0.644	0.770	0.383	0.447	0.413
	Concat	0.823	0.839	0.535	0.798	0.641
RENT	CFG	0.703	0.601	0.207	0.836	0.332
	OP	0.885	0.867	0.469	0.909	0.619
	SC	0.775	0.722	0.278	0.845	0.418
	Concat	0.876	0.871	0.476	0.881	0.618
TimeO	CFG	0.703	0.601	0.207	0.836	0.332
	OP	0.885	0.867	0.469	0.909	0.619
	SC	0.775	0.722	0.278	0.845	0.418
	Concat	0.876	0.871	0.476	0.881	0.618

**Table 8:** The multimodal decision fusion results.

Type	Sampling	AUC	ACC	P	R	F1	Weights
ARTHM	0	0.843	0.904	0.834	0.808	0.82	7.663, −2.486, 1.420, 4.740
	1	0.834	0.916	0.779	0.853	0.814	3.622, −1.596, 0.018, 4.876
LE	0	0.816	0.908	0.729	0.689	0.708	8.361, 0.390, 1.643, 2.933
	1	0.825	0.895	0.556	0.788	0.652	4.483, −1.898, 2.285, 3.356
RENT	0	0.636	0.884	0.560	0.290	0.382	5.451, −1.134, 2.180, 2.675
	1	0.852	0.909	0.280	0.890	0.426	2.016, 1.390, 1.050, 5.184
TimeO	0	0.844	0.948	0.64	0.745	0.688	13.704, 3.089, 3.664, 1.858
	1	0.886	0.948	0.507	0.888	0.645	7.540, 0.767, 1.707, 1.727

## Data Availability

Not applicable.

## Appendix A. An Example of Smart Contract Source Code

1 pragma solidity ^0.4.23;
3 interface Registry {
4         function setAttributeValue
5         ( address who, bytes32 what, uint val ) external;
6         function hasAttribute ( address _who, bytes32 _attribute )
7         external view returns ( **bool** );}
9 contract DepositAddressRegistrar {
10         Registry **public** registry;
11         bytes32 **public** constant IS_DEPOSIT_ADDRESS
12         = "isDepositAddress";
13         event DepositAddressRegistered
14         ( address registeredAddress );
15         constructor ( address _registry ) **public** {
16                 registry = Registry ( _registry );}
18 function registerDepositAddress (  ) **public** {
19                 address shiftedAddress = address ( uint( msg.sender ) >> 20 );
20                 require ( !registry.hasAttribute
21                 ( shiftedAddress , IS_DEPOSIT_ADDRESS ),
22                 "deposit address already registered" );
23                 registry.setAttributeValue
24                 ( shiftedAddress , IS_DEPOSIT_ADDRESS, uint( msg.sender ) );
25                 emit DepositAddressRegistered ( msg.sender );}
27 function(  ) external payable {
28                 registerDepositAddress (  );
29                 msg.sender.transfer ( msg.value );}}
